# Unqualified Dental Practice

**Published:** 1901-03

**Authors:** 


### UNQUALIFIED DENTAL PRACTICE.

Two deaths from septicæmia following close upon one another emphasize the danger to which the public are exposed in the existing state of the law. One which occurred in Manchester is instructive in several respects. The patient, a healthy girl, died of rapid septicæmia after the extraction of a tooth, notwithstanding that every effort was made to save her by the medical man attending her, and by a surgeon called in in consultation. The operation was performed at the establishment carried on by the executors of the late Mr. Parkinson, by an unqualified practitioner. It has long been known that in their advertisements the words "Exors. of" were inconspicuous, while the name of Mr. Parkinson was in large type, and it was elicited in evidence that the words "qualified by examination" appeared upon the place. The operator said that these words did not apply to him, and the jury, in asking the coroner to censure the executors, said that they published themselves as fully qualified dentists when there was not one about the place. Thus the public are deliberately deceived, and this has been going on for years.

In the other case the patient, who was found on a post-mortem to have died of septic meningitis, had gone to a chemist in Rotherhithe to have a tooth extracted, which operation was done by an unqualified person, and, according to the evidence of the surgeon who made the post-mortem, unskilfully, unnecessary injury having been inflicted. There was not the smallest doubt that the infection was caused at the time of the operation, and it is noteworthy that the patient had observed to his wife that the instrument was so dirty that he had nearly told the man not to put such a dirty thing into his mouth. It was produced at the inquest, and one juror remarked that it was more fit for drawing a horse's tooth than a man's, and the coroner noticed that it was still discolored.

Meanwhile, no opportunity should be neglected of giving the utmost publicity to such disasters as may occur, and very much may be done in this direction by individuals. Every dentist should avail himself of the opportunities afforded in his daily practice of talking to his patients about such cases as these here recorded; consistent effort in such directions will do more to educate public opinion than any amount of writing in the press.—*Abstract Editorial, Journal of the British Dental Association.*

